
JOURNAL INFORMATION
==============================
NLM Title Abbreviation: Innov Surg Sci
Journal ID: Innov Surg Sci
NLM Journal ID: 101708165
Journal ID: iss

Innovative Surgical Sciences

EISSN: 2364-7485
Publisher: De Gruyter

ARTICLE INFORMATION
==============================
PMCID: PMC13068489
DOI: 10.1515/iss-2026-2007
Article ID: iss-2026-2007
Article version: 1
Subjects: Article

7. Plastic, Reconstructive and Cosmetic Surgery

Electronic publication date: 2026 Apr 10
Publication date: 2026 Apr
Volume: 11
Issue: Suppl1
First page: 411
Last page: 420
Copyright: © 2026 by Walter de Gruyter Berlin/Boston
Copyright year: 2026
Copyright holder: De Gruyter
License: This work is licensed under the Creative Commons Attribution 4.0 International License.
License URL: https://creativecommons.org/licenses/by/4.0/

Page count: 10

==============================

Abstracts – DCK 2026 – 143rd Congress of the German Society of Surgery – Leipzig, April 22–24 • DOI 10.1515/iss-2026-2007
Innov Surg Sci 2026; 11 (Special Suppl 1): s411–s420

DE GRUYTER BRILL

**New Red Flags for Aesthetic Surgery: "Snapchat Dysmorphia"**
ID: E-DCK-241

**Plastische, Rekonstruktive und Ästhetische Chirurgie / Plastic, Reconstructive and Cosmetic Surgery**
**Ästhetische Chirurgie – operative Strategien & nicht-operative Verfahren / Cosmetic Surgery – Surgical Techniques and Non-Surgical Procedures**

Lena Busch[1 2 3], Haisam El Djabri[4], Fabian Tycho Nehls[1 5 6], Miriam Bogusch[1 2 5], Andre Dennes[7], Tobias Hirsch[1 2 5], Sonja Dahmann[1 2 5]

[1]Institut für Muskuloskelettale Medizin, Universität Münster, Plastische und Rekonstruktive Chirurgie, Münster, Deutschland, [2]Fachklinik Hornheide, Plastische-, Rekonstruktive- und Ästhetische Chirurgie, Handchirurgie, Münster, Deutschland, [3]Psychotherapeutische Privatpraxis, Münster, Deutschland, [4]Medizinisches Versorgungszentrum Hochsauerland, Praxis für Neurochirurgie und Neurologie, Arnsberg, Deutschland, [5]Klinik für Plastische Chirurgie, Universitätsklinikum Münster, Münster, Deutschland, [6]Klinik Dr. Hilpert, Duisburg, Deutschland, [7]Institut für Ausbildung und Studienangelegenheiten, Universität Münster, Münster, Deutschland

**Background:**
With the expanding opportunities for digital self-expression on social media, approaches to self-enhancement have also evolved. Photo filters enable the creation of images that extend well beyond the effects achievable with makeup or even plastic surgery. At the same time, persistent preoccupation with cosmetic concerns and constant comparison with unrealistic ideals may become pathological, as exemplified by body dysmorphic disorder (BDD). BDD is considered a major contraindication for aesthetic surgery and is characterized by a pathological preoccupation with appearance, attentional and evaluative biases toward one's own physical features, and the fact that these concerns cannot be "resolved" by surgical intervention. Accordingly, "red flags" related to unrealistic expectations shaped by digital photo-filter manipulation of appearance should be recognized by practitioners. In this study, we sought to investigate the so-called phenomenon of "Snapchat dysmorphia" as a potential new disease entity by examining the associations between social media use, photo-filter application, and symptoms of BDD.

**Material and methods:**
A total of N = 228 healthy participants completed online questionnaires. Social media and photo filter use, including self-reported motivations for filter application, were assessed. In addition, BDD symptoms—specifically perceived disfigurement, emotional burden, preoccupation with a potential flaw, and frequency of checking behavior—were measured according to current diagnostic criteria for BDD. Correlation and linear regression analyses were conducted using R statistical software.

**Results:**
We identified both overlaps and distinctions between BDD symptoms and the use of social media and photo filters. On the one hand, BDD symptoms appeared to be largely independent of social media engagement or photo filter use. On the other hand, the frequency of checking behavior was associated with both BDD symptomatology and social media/filter use. Furthermore, our analyses indicated that

Open Access. © 2026 The Author(s), published by De Gruyter. This work is licensed under the Creative Commons Attribution 4.0 International License.

photo filter use was linked to an increased tendency to strive for a “perfect,” and thereby unrealistic, physical appearance.

**Conclusion:**
The increasing trend toward digital beautification through photo filter use appears to be associated with unrealistic expectations regarding physical appearance as well as heightened checking behavior. In particular, we identified novel “red flags” for aesthetic surgery, namely exaggerated or unrealistic expectations of surgical outcomes in the context of social media photo filter use. However, we did not observe a pathological pattern in the sense of BDD that was directly attributable to photo filter use. Whether this phenomenon represents a pathological condition or a broader cultural shift in shaping surgical demand remains uncertain. However, when patients present digitally filtered images as reference points, this should be regarded as a clinical “red flag.” Such expectations often reflect unattainable ideals and may predispose to postoperative dissatisfaction. For cosmetic surgeons, these cases require careful preoperative counseling, clear communication of achievable outcomes, and, where necessary, consideration of psychological assessment.

DE GRUYTER BRILL

**Correlation Between Area Deprivation Index and Surgical Complications Following Mastectomy**
ID: E-DCK-91

**Plastische, Rekonstruktive und Ästhetische Chirurgie / Plastic, Reconstructive and Cosmetic Surgery**
**Ästhetische Chirurgie – operative Strategien & nicht-operative Verfahren / Cosmetic Surgery – Surgical Techniques and Non-Surgical Procedures**

Lioba Huelsboemer[1 2], Felix J. Klimitz[1], Fortunay Diatta[1], Laetitia Chiarella[1 3], Sam Boroumand[1], Alejandro Kochen[1], Alexander J. Kammien[1], Seungju Jackie Oh[1], Martin Kauke-Navarro[1], Bohdan Pomahac[1]

[1]Yale University - Division of Plastic Surgery, New Haven, Vereinigte Staaten, [2]Sana Klinikum Offenbach, Department of Burn, Hand, Plastic and Reconstructive Surgery, Offenbach, Deutschland, [3]Division of Plastic Surgery, Department of Trauma, Hand and Reconstructive Surgery, University Hospital Muenster, Muenster, Germany, Münster, Deutschland

**Background:**
The Area Deprivation Index is used by the American government to assess socioeconomic standing and persons at risk.This study investigates the association between high Area Deprivation Index values and post-surgical outcomes in patients undergoing mastectomy procedures.

**Material and methods:**
This retrospective cohort study examines the relationship between the Area Deprivation Index and postoperative outcomes among 767 patients who underwent mastectomy procedures within the Yale Healthcare Network, as recorded in the National Surgical Quality Improvement Program. Patients were excluded if data were missing for adjusting variables. Logistic regressions adjusted for age, body mass index, and race/ethnicity were performed to assess the association between Area Deprivation Index and surgical complications, unplanned 30-day readmission, and hospital length of stay.

**Results:**
Area Deprivation Index was tertiated to compare outcomes across different socioeconomic strata. Patients in the highest tertial had higher body mass index, were predominantly from historically minoritized backgrounds, and more frequently state-insured. They also exhibited a higher incidence of insulin-dependent diabetes and medicated hypertension. Higher Area Deprivation Index scores were significantly associated with a 50% increased risk of surgical complications (p=0.005). This effect persisted when adjusting for age and body mass index. However, when adjusted for race and ethnicity, the significance of the results diminished (p=0.106). Further, no significant associations were found for unplanned 30-day readmissions (p=0.33) or hospital length of stay (p=0.94).

**Conclusion:**
Our data indicate that individuals in higher Area Deprivation Index tertials are more susceptible to surgical complications. These findings highlight the need for targeted interventions and enhanced support mechanisms for economically disadvantaged populations to improve surgical outcomes and overall health.

DE GRUYTER BRILL

**Subcutaneous Fat Microbiome and Antimicrobial Peptides: a source for anti-infective therapies?**
ID: E-DCK-432

**Plastische, Rekonstruktive und Ästhetische Chirurgie / Plastic, Reconstructive and Cosmetic Surgery**
**Experimentelle Forschung: CTA und regenerative Grundlagenforschung / Experimental Research: Basic Research in Regenerative Medicine and CTA**

Nura Ahmad[1], Lukas Prantl[1], Andreas Eigenberger[1]

[1]University Hospital Regensburg, Department of Plastic, Hand and Reconstructive Surgery, Regensburg, Deutschland

**Background:**
The subcutaneous white adipose tissue has so far been little studied with regard to its microbiome and the antimicrobial properties of secreted proteins such as the cathelicidin antimicrobial peptide (CAMP). Such proteins could play an important role in inhibiting the growth of microbiological cell cultures.

**Material and methods:**
Skin and subcutaneous fat biopsies were collected under sterile conditions and analyzed microbiologically (BD-BACTEC, MALDI-TOF, susceptibility testing). Lipoaspirates were processed into three fractions (liquid portion, PBS wash, ALE), and LL-37 concentrations were quantified using ELISA. Primary endpoints included detection rate differences between skin and fat, microbial spectrum comparison, and correlation of LL-37 levels with bacterial growth in subcutaneous tissue.

**Results:**
Bacteria were detected in approx. 31.6 % of subcutaneous fat samples, with *S. epidermidis* predominating, while approx. 17.5 % showed bacterial growth exclusively in fat despite sterile skin, indicating a distinct subcutaneous microbiome. LL-37 was enriched in the ALE fraction.

**Conclusion:**
Viable bacteria were detected in 31.6 % of subcutaneous fat samples, partly independent of skin findings, indicating a distinct adipose microbiome. LL-37 was markedly enriched in the ALE fraction and reliably quantified. These findings reveal the dual metabolic and immune role of adipose tissue and suggest its potential as a source for autologous anti-infective therapies based on adipose-derived antimicrobial peptides.

Abstracts – DCK 2026 – 143rd Congress of the German Society of Surgery – Leipzig, April 22–24 • DOI 10.1515/iss-2026-2007
Innov Surg Sci 2026; 11 (Special Suppl 1): s411–s420

DE GRUYTER BRILL

**Chronic Rejection and Vasculopathy in Face Transplant Recipients**
ID: E-DCK-90

**Plastische, Rekonstruktive und Ästhetische Chirurgie / Plastic, Reconstructive and Cosmetic Surgery**
**Experimentelle Forschung: CTA und regenerative Grundlagenforschung / Experimental Research: Basic Research in Regenerative Medicine and CTA**

Lioba Huelsboemer[1 2], Martin Kauke-Navarro[1], Sam Boroumand[1], Neil Parikh[1], Helia Hosseini[1], Viola A. Stögner[1], Christine Ko[3], Richard N. Formica[4], Peter Hung[5], Amit Mahajan[5], Jamil R. Azzi[6], George F. Murphy[7], Bohdan Pomahac[1]

[1]Yale University - Division of Plastic Surgery, New Haven, Vereinigte Staaten, [2]Sana Klinikum Offenbach, Department of Burn, Hand, Plastic and Reconstructive Surgery, Offenbach, Deutschland, [3]Yale School of Medicine, Departments of Dermatology and Pathology, New Haven, Connecticut, USA, New Haven, Vereinigte Staaten, [4]Yale School of Medicine, Department of Medicine, Section of Nephrology and Transplantation, New Haven, CT, USA, New Haven, Vereinigte Staaten, [5]Yale School of Medicine, Department of Radiology and Biomedical Imaging, New Haven, Connecticut, USA, New Haven, Vereinigte Staaten, [6]Transplant Research Center, Renal Division, Brigham and Women's Hospital, Boston, MA, USA, Boston, Vereinigte Staaten, [7]Program in Dermatopathology, Department of Pathology, Brigham and Women's Hospital, Harvard Medical School, Boston, MA, USA, Boston, Vereinigte Staaten

**Background:**
Vasculopathy could lead to ischemia and potentially contribute to chronic rejection in allografts. However, the long-term assessment of chronic rejection and vasculopathy in face transplant recipients has been reported infrequently. This study aims to analyze the long-term occurrence of vasculopathy in the facial artery (allograft anastomosis) as indicator for chronic rejection.

**Material and methods:**
We retrospectively analyzed 35 computed tomography angiography scans from nine patients who underwent face transplantation at Brigham and Women´s Hospital (Boston, USA) between 2009 – 2020 and are now followed at Yale New Haven Hospital (New Haven, USA). The arterial diameter of proximal allograft vessels was serially measured as two orthogonal cross-sectional diameters using dynamic three-dimensional reconstructions at two index locations: just distal to the surgical anastomosis and at the facial artery near the submandibular gland. Measurements were performed utilizing Visage (version 7.1.18, San Diego, California). Three-dimensional volumetric reconstructions were generated using Syngo via Cinematic volume rendering (version 8.4, Siemens). Chronic rejection was assessed utilizing skin samples (H&E staining) through the proposed criteria from the American Society of Reconstructive Transplantation and International Society of vascularized composite allotransplant chronic rejection working group. Ordinary one-way ANOVA was used to analyze statistical significance between medians of cross-sectional surface area ($mm^2$).

**Results:**
The median follow-up was 120 months (range 54 – 154 months).

Clinical signs of chronic rejection were observed in three patients (33.3%). Histopathologic signs of chronic rejection were seen in four patients (44.4%). One patient (11.1%) underwent facial re-transplantation 88 months after the initial transplantation in the setting of irreversible allograft loss due to chronic antibody mediated rejection and repeated
acute cellular mediated rejection episodes that led to small-medium vessel graft arterial vasculopathy (deep vessels) resembling arteriosclerosis and graft loss. No significant decrease in distal facial arterial caliber was observed and no signs of vasculopathy (e.g. intima proliferation, stenosis) were detected in facial arteries.

**Conclusion:**
The long-term outcomes revealed promising results in terms of graft retention. Although, chronic rejection was observed in multiple patients, and led to irreversible allograft loss in one patient due to antibody-mediated rejection, no signs of vasculopathy were detected throughout the whole cohort in their facial arteries.

DE GRUYTER BRILL

**Redefining Surgical Treatment of Advanced Lymphedema: Liposuction-Assisted Excision**
ID: E-DCK-25

**Plastische, Rekonstruktive und Ästhetische Chirurgie / Plastic, Reconstructive and Cosmetic Surgery**
**Robotik & lymphatische Rekonstruktion / Robotics and Lymphatic Reconstruction**

Uzay Cambaz[1], Okan Gürsoy[2], Tahsin Acartürk[1]

[1]University of Pittsburgh, Department of Plastic Surgery, Pittsburgh, Vereinigte Staaten, [2]Istanbul University, Istanbul Faculty of Medicine, Istanbul, Turkey

**Background:**
Lymphedema is a chronic and progressive disease that primarily affects the extremities. In advanced stages, conservative measures are often insufficient, and surgical therapies become the focus of treatment. This study presents a surgical technique for managing severe, predominantly soft-tissue–dominated lymphedema of the lower extremity, which combines staged liposuction with functional excision. The aim of this method is to reduce limb volume, remove redundant skin, improve function, and minimize postoperative complications.

**Material and methods:**
Nine female patients with therapy-resistant lymphedema of the lower extremity were included. Seven had unilateral and two had bilateral disease. All had a history of oncological surgery followed by radiotherapy. The mean age was 58 years. Preoperatively, limb circumferences were measured at standardized anatomical points. The procedure began with staged tumescent liposuction in four defined zones, followed by excision of the tissue via a medial longitudinal incision. Skin and subcutaneous tissue were reshaped and prepared for primary tension-free wound closure. Drains were placed in selected cases. Postoperatively, compression therapy was applied for four weeks, followed by eight weeks of physiotherapy. From the third postoperative month, flat-knitted compression garments were fitted. In cases of bilateral disease, the more severely affected limb was operated on first, with the second procedure performed after an interval of six months. In total, 11 limbs were treated. The aspirated fat volume and excised skin area were documented. One year after surgery, limb circumferences were re-measured at standardized points and compared with baseline values.

**Results:**
In two cases, minor wound dehiscence occurred, which was managed conservatively. No seromas or infections were observed. All patients reported a marked improvement in quality of life and daily functioning. Limb circumferences were significantly reduced. No recurrences or late infections were noted during the follow-up period of more than one year. In addition to the reduction in limb volume, a general weight loss was observed. Some patients also reported volume reduction in non-operated regions, including the contralateral limb and proximal thighs. Overall quality of life improved significantly in all patients.

DE GRUYTER BRILL

**Conclusion:**
In advanced, predominantly soft-tissue–dominated lymphedema, the combination of liposuction and excisional surgery represents a functional, low-morbidity, and aesthetically superior alternative to classical procedures such as the Charles or Homans operation. In contrast to isolated liposuction, the simultaneous excision allows for the removal of redundant skin and helps avoid typical complications such as seromas or necrosis. Moreover, prior liposuction facilitates the mobilization of flap tissue and enables safer resection. In carefully selected patients, this combined approach can reduce complication rates while sustainably improving patient satisfaction.

Abstracts – DCK 2026 – 143rd Congress of the German Society of Surgery – Leipzig, April 22–24 • DOI 10.1515/iss-2026-2007
Innov Surg Sci 2026; 11 (Special Suppl 1): s411–s420

DE GRUYTER BRILL

### The Book-Cover Flap: A Novel Solution for Massive Localized Lymphedema of the Medial Thigh

ID: E-DCK-34

**Plastische, Rekonstruktive und Ästhetische Chirurgie / Plastic, Reconstructive and Cosmetic Surgery**
**Robotik & lymphatische Rekonstruktion / Robotics and Lymphatic Reconstruction**

Uzay Cambaz[1], Okan Gürsoy[2]

[1]University of Pittsburgh, Department of Plastic Surgery, Pittsburgh, Vereinigte Staaten, [2]Istanbul University, Istanbul Faculty of Medicine, Istanbul, Turkey

**Background:**
Massive localized lymphedema (MLL) is a rare but progressive disorder characterized by extensive soft-tissue overgrowth due to impaired lymphatic drainage. It occurs most frequently in severely obese patients and typically involves the medial thighs, lower abdomen, and groin. MLL leads to significant hygienic and functional limitations, recurrent infections, and a significantly reduced quality of life. Conservative therapies are usually ineffective in the long term, leaving surgical excision as the only practical option. However, classical elliptical excision techniques, as frequently described in the literature, are associated with high complication rates, including wound dehiscence, infection, and excessive tissue tension.
In this study, we present a novel approach for excision planning and defect closure in medial thigh MLL: functional excision followed by reconstruction with a posterior-based fasciocutaneous “Book-Cover Flap.”

**Material and methods:**
Surgical technique: With the legs positioned in abduction, the MLL mass of the medial thigh was placed freely on the operating table. The anterior and medial borders of the lesion were included in the planned resection, while the posterior portion was designed as a posterior-based fasciocutaneous flap for later reconstruction. After skin incision, the lesion was separated from the thigh using a bimanual “cracking” technique. The deep fascia was preserved in situ to control bleeding and protect neurovascular structures. The resulting defect was then closed in a tension-free manner using the posterior-based flap in a “book-cover” fashion, with multilayer adaptation. All specimens were submitted for histopathological analysis.

**Results:**
A total of 11 patients were treated (7 unilateral, 4 bilateral MLL). In all cases, primary tension-free closure was achieved in a single stage. No flap necrosis, wound dehiscence, or major healing complications occurred. No contour deformities or differences in skin texture were observed in the reconstructed thigh. Long-term follow-up revealed neither recurrence of lymphedema nor development of angiosarcoma in the surgical area.

**Conclusion:**
Functional excision combined with reconstruction using the “Book-Cover Flap” represents a practical, safe, and effective surgical solution for closing significant medial thigh MLL defects. Compared to conventional techniques, this method offers several clear advantages: lower complication rates due to reduced tissue tension, durable soft-tissue coverage, and aesthetically and functionally convincing results without contour deformities. This innovative approach has the potential to redefine the standards for reconstructive management of massive localized lymphedema.
